# Impact of indirect transitions on valley polarization in WS_2_ and WSe_2_†

**DOI:** 10.1039/d2nr04800k

**Published:** 2022-11-21

**Authors:** Rasmus H. Godiksen, Shaojun Wang, T. V. Raziman, Jaime Gómez Rivas, Alberto G. Curto

**Affiliations:** Dep. Applied Physics and Institute for Photonic Integration, Eindhoven University of Technology Eindhoven The Netherlands A.G.Curto@TUe.nl; MOE Key Lab. of Modern Optical Technologies and Jiangsu Key Lab. of Advanced Optical Manufacturing Technologies, School of Optoelectronic Science and Engineering, Soochow University Suzhou 215006 China; Photonics Research Group, Ghent University-imec Ghent Belgium; Center for Nano- and Biophotonics, Ghent University Ghent Belgium

## Abstract

Controlling the momentum of carriers in semiconductors, known as valley polarization, is a new resource for optoelectronics and information technologies. Materials exhibiting high polarization are needed for valley-based devices. Few-layer WS_2_ shows a remarkable spin-valley polarization above 90%, even at room temperature. In stark contrast, polarization is absent for few-layer WSe_2_ despite the expected material similarities. Here, we explain the origin of valley polarization in both materials based on the interplay between two indirect optical transitions. We show that the relative energy minima at the Λ- and K-valleys in the conduction band determine the spin-valley polarization of the direct K–K transition. Polarization appears as the energy of the K-valley rises above the Λ-valley as a function of temperature and number of layers. Our results advance the understanding of the high spin-valley polarization in WS_2_. This insight will impact the design of both passive and tunable *valleytronic* devices operating at room temperature.

## Abstract 2

Valley polarization is a new resource for optoelectronics and information technologies. This article demonstrates the critical role of indirect transitions in protecting valley polarization in few-layer WS_2_ and WSe_2_.

## Graphical abstract

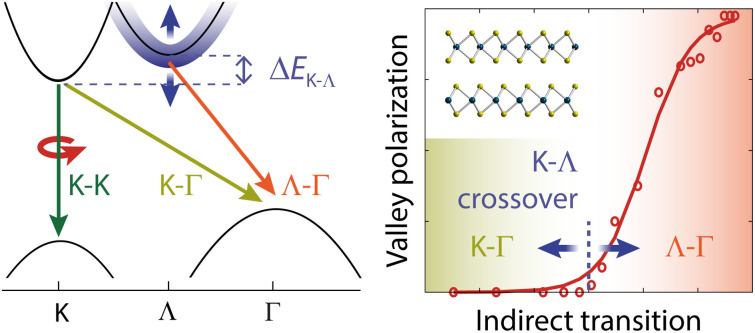

Transition metal dichalcogenides (TMDs) such as MoS_2_, WS_2_, or WSe_2_ are layered semiconductors with unique spin-valley physics. The coupling between spin and momentum for excited carriers opens a new path to access the valley degree of freedom.^1^ Valley polarization arises from a difference in exciton populations at the K- and K′-points of the hexagonal Brillouin zone (Fig. 1a),^1–3^ where local energy minima known as valleys lie. At these diametrically opposite points in reciprocal space, strong spin–orbit splitting occurs in the top valence bands. The different signs of the splitting in the K- and K′-valleys^4^ result in the coupling of spin and valley indexes and leads to spin-dependent optical and electronic properties. The K- and K′-valleys can be selectively excited using right- or left-handed circularly polarized light.^5^ TMDs constitute thus a fascinating platform for future *valleytronic*,^6^ optoelectronic,^7–9^ and nanophotonic^10,11^ devices exploiting the spin, valley, and layer indexes.

**Fig. 1:**
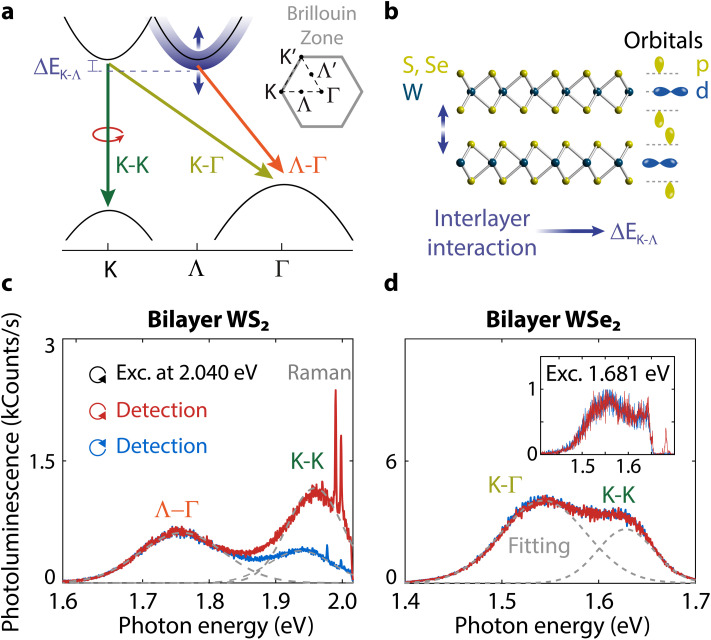
Direct and indirect optical transitions in few-layer TMDs and their spin-valley polarization. (a) Possible exciton transitions in few-layer WS_2_ and WSe_2_. Two indirect transitions can occur depending on whether the conduction band minimum is either at the K- or the Λ-valley. Their relative energy difference is Δ*E*_K–Λ_. Intervalley K–K′ scattering is omitted for clarity. (b) Illustration of a bilayer WS_2_ or WSe_2_ and the orbital characters of the K- and Λ-valleys. The interlayer interactions through the p-orbitals control the Λ-point energy resulting in changes of Δ*E*_K–Λ_. Polarization-resolved photoluminescence spectra for (c) bilayer WS_2_, and (d) bilayer WSe_2_ excited with photon energy 2.040 eV at room temperature. There is high polarization in WS_2_ and no polarization in WSe_2_. Inset: lack of polarization for bilayer WSe_2_ when excited with 1.681 eV close to resonance to its K–K transition.

The layered nature of TMDs enables a high degree of control over valley polarization. A monolayer possesses a direct band gap, whereas in the few-layer regime the band gap becomes indirect.^12–14^ Light emission in few-layer TMDs is dominated by indirect transitions from the Λ- and K-points to the Γ-point in the band structure (Fig. 1a). These indirect transitions are typically unpolarized. At higher energy, polarized intravalley transitions with direct character can still occur in the K- or K′-valleys.^15^ The degree of circular polarization can be used as a proxy for valley polarization. It is defined as DOCP = (*I*_σ+_ − *I*_σ−_)/(*I*_σ+_ + *I*_σ−_), where *I*_σ+_ and *I*_σ−_ are the photoluminescence intensities with right- and left-handed circular polarization, respectively. Valley polarization can reach values near unity at cryogenic temperatures for monolayer MoS_2_.^16^ With increasing temperature, however, the initial polarization quickly depolarizes due to intervalley scattering between the K- and K′-points,^17^ limiting applications at room temperature. At higher temperatures, a valley polarization enhancement for a monolayer has been realized through interaction with graphene,^18,19^ reaching up to 40% DOCP for graphene-encapsulated WS_2_.^20^ In contrast to the monolayer case, the DOCP reaches 65% for bilayer WS_2_ even at room temperature.^21^ Such a large spin-valley polarization in bilayer WS_2_ is not well understood yet.^22,23^

Despite sharing several properties with WS_2_ due to the common W atom, valley polarization is absent in WSe_2_ at room temperature. This discrepancy between bilayer WS_2_ and WSe_2_, illustrated in Fig. 1c and d, is inconsistent with theoretical predictions.^15^ Hence, it remains unresolved, as stated by Bussolotti *et al.*^22^ An understanding of the spin-valley properties that lead to high and low polarization in WS_2_ and WSe_2_ is, therefore, essential for practical applications at room temperature.^24^ Gaining insight into the spin-valley physics of bilayer TMDs would also be beneficial for spin-layer locking effects,^25–28^ layer-dependent spin relaxation,^29^ and the spin-valley Hall effect in few-layer systems.^30,31^

Here, we demonstrate the critical role of the Λ-valley on the spin-valley polarization in few-layer WS_2_ and WSe_2_ through a combined investigation of polarization- and temperature-resolved photoluminescence (PL). By varying both the number of layers and the temperature, we analyze the interplay between the momentum-allowed direct transition (K–K) and two momentum-forbidden indirect transitions (K–Γ and Λ–Γ). We find that a change in the dominant indirect transition channel with temperature determines the observation of spin-valley polarization. In bilayer WSe_2_, we reveal the existence of a crossover temperature at which the dominant indirect transition switches from K–Γ to Λ–Γ as the Λ-point energy shifts lower in energy than the K-point. Below this crossover temperature, the polarization of the direct K–K transition begins to increase even for highly off-resonant excitation. We demonstrate the dependence of the valley polarization of the direct K–K transition on the K–Λ energy difference in the conduction band. In contrast to WSe_2_, the Λ–Γ indirect transition dominates the emission in WS_2_ resulting in high polarization even at room temperature. Based on our results, we explain how both temperature and number of layers affect spin-valley polarization in WS_2_ and WSe_2_. Therefore, we identify a missing piece of the puzzle for understanding and achieving high spin-valley polarization in few-layer semiconductors.

## Experimental methods

### Sample fabrication

We deposit WS_2_ and WSe_2_ microcrystals onto SiO_2_/Si (285 nm thick SiO_2_) substrates by mechanical exfoliation from synthetic, bulk 2H crystals (HQ Graphene). We first determine the thickness of the flakes by optical contrast microscopy^32^ and by considering the energy of the indirect exciton emission in photoluminescence spectra.^33^ After optical measurements, we confirmed the thickness by atomic force microscopy.

### Optical measurements

We carry out photoluminescence measurements using a microscope in *epi*-fluorescence geometry (objective lens: Nikon CFI Plan Fluor ELWD 40×, NA = 0.6). We typically excite the microcrystals with a continuous-wave laser with photon energy 2.040 eV and a power of 12.2 μW before the objective lens, ensuring a power density in the linear response range for the TMDs. For WSe_2_, we also used a continuous-wave 1.796 eV laser with a power of 75.6 μW. For one measurement with bilayer WSe_2_, we used a supercontinuum laser (Fianium SC400, pulse duration ∼50 ps) with an acousto-optical filter tuned to 1.681 eV and 1.7 μW. To control the circular polarization in excitation, we employ a Babinet–Soleil compensator and a Stokes polarimeter (PolSNAP, Hinds Instruments) at the sample position to ensure circular polarization of the incident laser beam. In the detection path, we use a non-polarizing beamsplitter (21 014 silver non-polarizing 50/50 bs, Chroma), and then either two 615 nm longpass filters (ET615LP Chroma), two 700 nm longpass filters (FELH0700, Thorlabs), or one 750 nm longpass filter (FELH0750, Thorlabs). For emission polarization analysis, we combine a quarter-wave plate (achromatic quarter-wave retarder, 600–1200 nm, Bernhard Halle) and a wire-grid polarizer (WP25M-UB, Thorlabs). After coupling into an optical fiber with core size 50 μm, we record PL spectra with an Andor Shamrock 303i spectrometer and an Andor Newton 970 EMCCD camera. For low-temperature measurements, we use a liquid helium flow cryostat (Oxford Instruments MicrostatHiRes) pumped to ultra-high vacuum.

## Results and discussion

### Spin-valley polarization in few-layer WS_2_ and WSe_2_

First, we consider the typical band structure of a bilayer TMD (Fig. 1a). The bilayer band gap is indirect because the valence band maximum shifts from K to Γ from one to two layers. The nature of the indirect transition depends on the competition between the Λ and K conduction band energy minima (orange and light green arrows in Fig. 1a). To study the impact of these indirect transitions on polarization, we will exploit their dependence on temperature and number of layers to tune the band structure.

The effect of interlayer interactions on the band structure is highly dependent on momentum, leading to a different layer and temperature dependence for the energy of the K–K, K–Γ, and Λ–Γ transitions (Table 1).^17,34^ At the K-point, d-orbitals from the transition metals determine the top-most band structure.^5^ Increasing the temperature expands the covalent bond length between the atoms reducing the energy gap at the K-point. The transition metal atoms are protected between the chalcogens, which results in insensitivity of the K-point to the surrounding medium and, therefore, to the number of layers. On the other hand, the chalcogen atoms lie close to both the surrounding medium and the adjacent layers. The chalcogen p-orbitals that dominate at the Λ-point extend outside the atomic plane, rendering it sensitive to interlayer interactions (Fig. 1b).^34^ With increasing temperature, the out-of-plane p-orbitals extend in length and come closer to each other, thereby increasing their interaction because the interlayer distance due to van der Waals forces between the layers is not temperature dependent. Consequently, the Λ-valley increases in energy with increasing temperature. Increasing the number of layers, on the contrary, results in a decrease of the Λ-valley energy because more out-of-plane p-orbitals interact with neighboring layers. As summarized in Table 1, we can utilize both temperature and the number of layers to alter the direct and indirect transitions of WS_2_ and WSe_2_.

**Table 1:** The number of layers and temperature affect the band structure of few-layer WS_2_ and WSe_2_, resulting in different dependences for the transition energies between different points in momentum space

Energy	Increase in #*L*	Increase in *T*
*E*_K–K_	Near constant	Decreases
*E*_Λ–Γ_	Decreases	Increases
*E*_K–Γ_	Decreases	Decreases

To compare the valley polarization of WS_2_ and WSe_2_, we excite our samples with circularly polarized light and measure the polarization of the emission with a circular polarization analyzer and a spectrometer (see Experimental methods). We observe a stark difference in circular polarization for bilayer WS_2_ and WSe_2_ (compare high and low values in Fig. 1c and d) for excitation with laser photon energy of 2.040 eV close to resonance with WS_2_. We confirmed that the low polarization for WSe_2_ is not due to off-resonant excitation by using two additional excitation energies of 1.796 eV and 1.681 eV (Fig. 1d, inset, and ESI Fig. S1†). We still observed no polarization at room temperature despite having nearly the same detuning with the K–K emission of 66 meV in WSe_2_ excited by 1.681 eV compared to WS_2_ excited by 2.040 eV. In this work, we will demonstrate the dependence of the polarization in WS_2_ and WSe_2_ on the indirect band gap character controlled by the energy difference Δ*E*_K–Λ_ (Fig. 1a). To clarify the role played by Δ*E*_K–Λ_ on the differences and similarities between the polarization of WS_2_ and WSe_2_, we measure next the changes in spectra and polarization as a function of the number of layers and temperature.

### The role of the indirect optical transitions in polarization

**Fig. 2:**
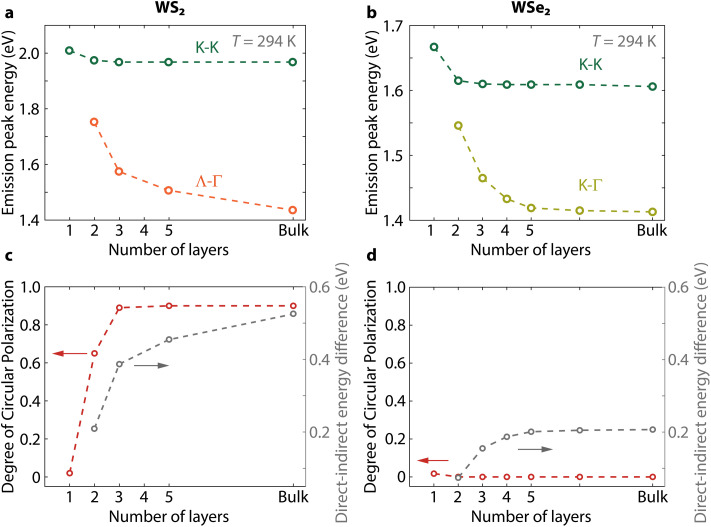
Different dependences of the spin-valley polarization of WS_2_ and WSe_2_ on the number of layers. (a and b) Photoluminescence peak positions of the transitions. (c and d) Circular polarization of the K–K band maximum for different numbers of layers of WS_2_ and WSe_2_, respectively, under excitation at 2.040 eV. See text for similar results for WSe_2_ excited close to resonance. We retrieve the polarization of the K–K transition using Gaussian fitting of all the spectra detecting each circular polarization in order to remove any possible spectral overlap with indirect transitions. The dashed lines are guides to the eye. All measurements at room temperature.

We prepare samples with varying numbers of layers of WS_2_ and WSe_2_. We measure their PL spectra and determine the position of the direct and indirect transition peaks (ESI Fig. S2†). The peak energies as a function of the number of layers show that the separation between the direct and indirect peaks increases faster with thickness for WS_2_ compared to WSe_2_ (Fig. 2a and b). This difference is a consequence of the origin of their indirect emission, which stems from Λ–Γ transitions in WS_2_ while it originates from K–Γ transitions in WSe_2_ at room temperature.^34^ The larger increase in energy shift with thickness for the Λ–Γ transition is due to the larger impact of interlayer interactions on the Λ-valley compared to the K-valley.

Next, we measure the change of polarization for a varying number of layers (Fig. 2c and d, and ESI Fig. S3†). For WS_2_, the polarization of the K–K transition quickly increases from mono- to trilayer, reaching a DOCP = 0.89 and saturating for thicker samples. For WSe_2_, the polarization of the K–K transition remains absent for all thicknesses even when exciting closer to resonance (ESI Fig. S4†). As expected, the K–Γ and Λ–Γ transitions are unpolarized in all measurements (ESI Fig. S2 and S3†). We deconvolute the polarization contribution of each transition by fitting the spectra with Gaussian functions (see Experimental methods). Thanks to this fit, we remove any contribution from the unpolarized indirect PL emission in our polarization analysis to retrieve the DOCP for the PL maximum of the direct transition alone.

**Fig. 3:**
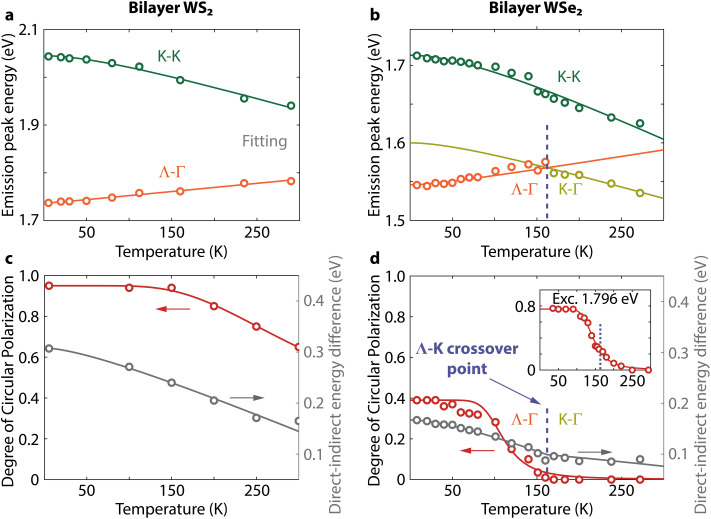
Relation between the indirect band gap character and spin-valley polarization. (a and b) Temperature dependence of the photoluminescence peak position for direct and indirect transitions. (c and d) Temperature dependence of the circular polarization of the K–K transition maximum for bilayer WS_2_ and WSe_2_, respectively. The polarization of the K–K transition in WSe_2_ starts increasing when the conduction band minimum shifts from K to Λ below the crossover temperature (blue dashed line). Inset: polarization under near-resonant excitation for WSe_2_. Solid lines are fits as described in the text.

The insensitivity of the polarization to thickness in WSe_2_ is in clear contrast to the dependence in WS_2_. As the main change in band structure with increasing thickness is a decrease in energy of the Λ–Γ transition (*E*_Λ–Γ_), we can reasonably expect that an increasing difference between *E*_K–K_ and *E*_Λ–Γ_ could determine the increase in circular polarization in WS_2_. To validate this hypothesis, however, we need to determine the conditions required for increasing the DOCP in WSe_2_. Changing the temperature is a controllable way to perturb the band structure in both materials. Thus, we measure next the PL spectra and DOCP at lower temperatures and track the PL peak positions (Fig. 3 and ESI Fig. S4†). In bilayer WS_2_, the direct and indirect exciton peaks move to higher and lower energies with decreasing temperature, respectively (Fig. 3a). In WSe_2_, the situation is different. First, the K–Γ peak shifts to higher energy with decreasing temperature because the K-point is the conduction band minimum in this temperature range.^34^ Below 160 K, the indirect peak starts moving to lower energies with decreasing temperature (Fig. 3b), which is consistent with the indirect peak now arising from Λ–Γ transitions.

We describe the evolution of the peak energies with temperature (Fig. 3a and b) using the Varshni equation:^35^1
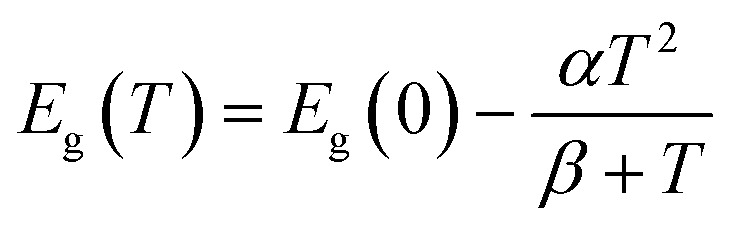
where *T* is the temperature, *E*_g_(0) is the excitonic band gap at *T* = 0 K, and *α* and *β* are phenomenological fitting parameters. For the indirect exciton in WSe_2_, we use two separate Varshni equations for the high- and low-temperature regimes due to the change in indirect transition character at *T* = 160 K. We list the fitting parameters in Table 2. The parameter *α* describes the band gap change with temperature due to thermal expansion of the lattice. The values of *α* for the K–K transition and the Λ–Γ transition are equal for both materials, demonstrating their similar dependence of band structure on temperature. However, bilayer WSe_2_ has a smaller band gap than WS_2_. As a result, the K–K and Λ–Γ transitions will be closer in energy in WSe_2_. Therefore, the crossover of the indirect transitions occurs at a lower temperature in WSe_2_ than in WS_2_.

**Table 2:** Fitting parameters using the Varshni equation for the temperature dependence of the different transitions and materials in Fig. 3a and b

Material/transition		*E*_*g*_(0) (eV)	*α* (meV K^−1^)	*β* (K)
WS_2_	K–K	2.045	0.530	118.9
	Λ–Γ	1.737	−0.172	12.5
WSe_2_	K–K	1.713	0.530	139.3
	K–Γ	1.600	0.316	96.6
	Λ–Γ	1.546	−0.172	44.1

The measured polarization rises with increasing number of layers in WS_2_ because it corresponds to a higher K–Λ energy separation of the conduction bands. Similarly, this energy separation also increases with decreasing temperature. Consequently, for bilayer WS_2_ the circular polarization increases with decreasing temperature as well (Fig. 3c). At temperatures from 300 to 160 K, valley polarization remains absent in WSe_2_ when excited off resonance at 2.040 eV. In this temperature range, the *E*_K–K_–*E*_K–Γ_ separation does not vary substantially because both peaks shift to higher energy with decreasing temperature (gray points in Fig. 3d). Below *T* = 160 K, the indirect transition changes from K–Γ to Λ–Γ. Simultaneously, the polarization of the K–K transition starts to increase and saturates at low temperatures (Fig. 3d, including higher polarization under near resonant excitation). At these low temperatures, bilayer WSe_2_ behaves like bilayer WS_2_ because their indirect transitions have now both Λ–Γ character, as evidenced by their similar Varshni dependences.

Our polarization values at low temperature (*T* ∼ 10 K) are consistent with previous measurements using similar excitation energies for both materials.^36,37^ Reaching a DOCP of 0.39 at 35 K in bilayer WSe_2_, despite exciting 320 meV away from the K–K transition demonstrates the critical role of the Λ-valley in establishing the robust spin-valley polarization in few-layer WS_2_ and WSe_2_.

To compare the temperature dependence of polarization in few-layer WS_2_ and WSe_2_, we fit the DOCP as a function of temperature (Fig. 3c and d) using the expression2
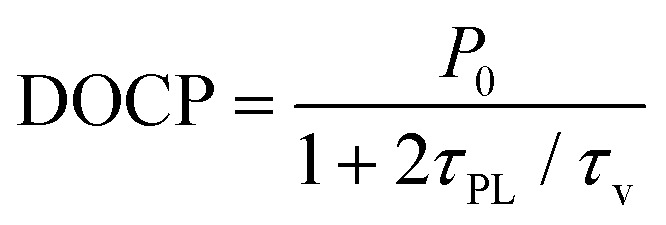
which takes into account the K–K exciton transition rate (1/*τ*_PL_) and the K–K′ intervalley scattering rate (1/*τ*_v_).^1^*P*_0_ is the initial polarization before scattering takes place, for which we use the maximum DOCP at the lowest measured temperature. We assume that excitons follow a Boltzmann distribution for the ratio *τ*_PL_/*τ*_v_ = *c* exp(−Δ*E*/*k*_B_*T*),^38^ where *c* is a constant and Δ*E* is an activation energy needed to undergo K–K′ intervalley scattering (see ESI Table S1† for fitting parameters).

**Fig. 4:**
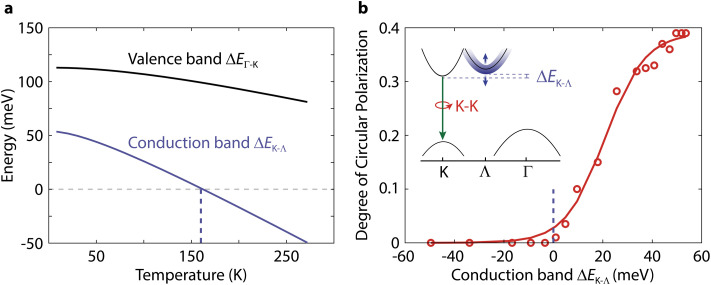
The polarization of the direct K–K transition in WSe_2_ depends on the energy difference between the conduction band points of the indirect transitions. (a) Temperature dependence of Δ*E*_K–Λ_ in the conduction band and Δ*E*_Γ–K_ in the valence band obtained from the three fits in Fig. 3b. (b) Polarization of the K–K photoluminescence excited with 2.040 eV as a function of the Δ*E*_K–Λ_ conduction band difference. Inset: schematic of the band diagram responsible for the changes in K–K polarization determined by the value of Δ*E*_K–Λ_.

Next, we explicitly demonstrate that the polarization of the direct K–K transition in WSe_2_ depends on the K–Λ energy difference in the conduction band, Δ*E*_K–Λ_. First, we retrieve Δ*E*_K–Λ_ as a function of temperature from fitting Fig. 3b as Δ*E*_K−Λ_ = *E*_K−Γ_ − *E*_Λ−Γ_ (Fig. 4a). The polarization of the K–K transition starts increasing when Δ*E*_K–Λ_ becomes positive (Fig. 4b). Previously, the Γ-hill has been suggested to be involved in K–K′ intervalley scattering by slowing down the scattering of holes from K to K′ in bilayer WS_2_ compared to monolayer WS_2_.^39^ That hypothesis is not consistent, however, with the absence of valley polarization in WSe_2_ at higher temperatures, where the dominant indirect transition is K–Γ. Instead, the Λ-valley could play a similar role in the scattering of electrons. A comparison of Δ*E*_K–Λ_ in the conduction band and Δ*E*_Γ–K_ in the valence band (Δ*E*_Γ−K_ = *E*_K−K_ − *E*_K−Γ_) further supports the relevance of the Λ-valley on polarization (Fig. 4a). The energy difference in the valence band Δ*E*_Γ–K_ is already far above the thermal energy at room temperature, so its weak increase at lower temperatures cannot influence polarization substantially. On the other hand, Δ*E*_K–Λ_ is similar to the thermal energy near the Λ–K crossover. Thus, excitons will populate both Λ–Γ and K–Γ states, resulting in the weaker polarization increase before the Λ–K crossover (Fig. 3d). Our results thus highlight the important role of the Λ-valley in protecting spin-valley polarization in few-layer semiconductors.

### Mechanisms for spin-valley polarization in few-layer semiconductors

**Fig. 5:**
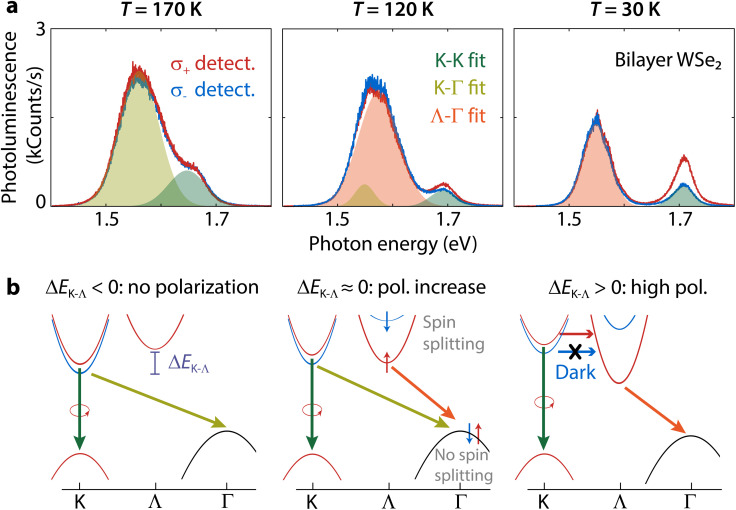
Effect of the K–Λ crossover on the K–K spin-valley polarization for bilayer WSe_2_. (a) Photoluminescence spectra and (b) band structure schematics illustrating the conditions leading to the appearance of polarization at three different temperatures. The emission spectra include fits to a Gaussian function for the detected σ_+_-polarization spectra. At *T* = 170 K (left column), there is no polarization and the Λ-valley is not yet involved in the transitions. At this temperature, the K–Γ transition can deplete the spin-down K-valley because there is no spin splitting in the Γ-hill. At *T* = 120 K (center), a small spin-valley polarization appears as the Λ-valley now takes part in the optical transitions. Here, two Gaussians are necessary for fitting the indirect photoluminescence peak. At *T* = 30 K (right), only the Λ-valley contributes to the indirect emission resulting in higher polarization. Due to spin splitting in the Λ-valley, the spin-down K-valley is no longer depleted through indirect optical transitions and becomes dark. All spectra were acquired using 2.040 eV excitation.

To better illustrate the appearance of polarization below the crossover temperature between indirect transitions, we depict how the band structure changes for three different temperatures (Fig. 5). When *T* > 160 K, the indirect transition is K–Γ and polarization is absent for the K–K transition (Fig. 5, left). As the temperature decreases, Λ moves to an energy similar to K. There is an intermediate temperature range where both K–Γ and Λ–Γ transitions contribute to the indirect emission and the K–K polarization starts to increase (Fig. 5, center). The overlap in emission from both indirect transitions is evident from the fitting of the indirect spectral band, where two Gaussians are necessary. Finally, as Δ*E*_K–Λ_ increases, only the Λ-valley contributes to indirect emission resulting in a faster increase of polarization with decreasing temperature (Fig. 5, right).

We discuss next how the Λ-valley could lead to a reduced intervalley scattering rate. We focus on two mechanisms for intervalley scattering and how they compare to our results:

(I) Intervalley scattering by phonons.^40–42^

(II) Intervalley scattering by the long-range exchange mechanism.^17,43,44^

The coupling between excitons and phonons can cause the spin to flip, but this process also requires phonons with the right momentum. A strong exciton–phonon coupling promotes spin-flipping.^45^ We extract the exciton–phonon coupling using the O'Donnell equation^46^ to fit the PL peaks in Fig. 3a and b. The results indicate that Λ–Γ excitons have a lower exciton–phonon coupling strength (ESI section S2.2†) and are thus the least likely to undergo intervalley scattering by phonons. Additionally, intervalley scattering by phonons can occur much faster for electrons due to the low spin splitting of the conduction band at the K-point enabling faster spin-flipping.^40^ Due to its lower energy and the very large spin splitting,^47^ the Λ-valley could introduce a very efficient ‘trap’ preventing electrons scattered by phonons from reaching the K′-valley. Thus, K–K′ intervalley scattering by phonons could be slowed down significantly when the Λ-valley is the conduction band minimum. Intervalley scattering by phonons is thus consistent with the polarization trends illustrated in Fig. 5.

Alternatively, the long-range exchange mechanism can cause a K–K exciton to undergo intervalley scattering by recombining and exciting an exciton at the K′-valley. Compared to phonons, it does not require any additional momentum. Since the process occurs more efficiently for excitons of higher kinetic energy, the Λ-valley slows down the K–K′ intervalley scattering rate by trapping K–K excitons of higher kinetic energy more efficiently. From the fitting of DOCP as a function of temperature with eqn (2), we note that the values of Δ*E* (ESI Table S1†) do not match with the phonon energy required for K–K′ intervalley scattering.^48,49^ Δ*E* could thus correspond better with the excess energy required by the long-range exchange mechanism. Nevertheless, such a simple fit likely does not take into account all of the temperature-dependent material parameters affecting the spin-valley polarization.^18^

Additionally, spin-forbidden dark excitons with lower energy than the bright excitons^50^ can increase polarization. Dark excitons have previously been attributed to the robust spin-valley polarization in monolayer tungsten systems with respect to both temperature and excitation energy.^51^ We demonstrate the presence of dark excitons in ESI section S2.3† and estimate a bright-dark splitting *E*_BD_ = 37.9 meV, which is agreement with the value in monolayer WSe_2_.^52^ As there is no K–K′ intervalley exchange interaction for dark excitons,^17^ they can act as a reservoir for the bright exciton valley polarization.^51^ On the other hand, the K–Γ transition depopulates the dark exciton reservoir contributing to depolarization because there is no spin splitting at Γ.^22^ This situation is consistent with the low polarization at temperatures above the Λ–K crossover (Fig. 5, left). In the temperature range where *E*_Λ_ < *E*_K_, the K–K dark exciton reservoir is restored due to the spin splitting at Λ,^22,47^ resulting in a high and robust polarization even for off-resonant excitation (Fig. 5, right).

Finally, we show for completeness that the dependence of polarization on the number of layers is also in agreement with the mechanism in Fig. 5 where polarization is controlled by Δ*E*_K–Λ_. For WS_2_, there is a significant difference in polarization between bilayers and trilayers at room temperature (DOCP ≈ 0.65 and 0.95 in Fig. 2c). The addition of one layer shifts the Λ-valley to lower energy, while the K-valley remains nearly constant (Fig. 2a), thus increasing Δ*E*_K–Λ_. To confirm this behavior in WSe_2_ as well, we perform temperature- and polarization measurements on WSe_2_ from one to four layers showing an increase of polarization with number of layers (ESI Fig. S7†). The results are consistent with the expectation of a smaller Δ*E*_K–Λ_ energy separation at elevated temperatures with increasing thickness, resulting in the Λ–K crossover occurring at a higher temperature. The polarization at *T* = 35 K is much higher in bilayer WSe_2_ excited off resonance than in monolayer WSe_2_ excited near resonance (DOCP ≈ 0.79 compared to 0.23) despite the additional detuning. This polarization difference between bilayer and monolayer WSe_2_ is consistent with the Λ-valley offering additional protection for K–K′ intervalley scattering by phonons in bilayers.

## Conclusion

In summary, we have demonstrated the impact of the Λ-valley on spin-valley polarization in WS_2_ and WSe_2_ through temperature- and polarization-resolved photoluminescence measurements. By varying the temperature and the number of layers, the position of the conduction band Λ-valley changes relative to the K-valley. We show that the conduction band Λ–K energy difference controls the K–K spin-valley polarization resulting in robust polarization. In bilayer WSe_2_, we correlate the appearance of polarization with the crossover between indirect transitions below *T* = 160 K, when the Λ-valley becomes the conduction band minimum. The polarization increases with the energy difference between the K- and Λ-valleys. This observation highlights the importance of the Λ-valley in blocking K–K′ intervalley scattering to stabilize polarization.

Our results introduce the critical role of indirect optical transitions in spin-valley polarization in few-layer semiconductors, contributing in particular to the understanding of the exceptionally high spin-valley polarization in few-layer WS_2_. For WS_2_, the energy of the Λ-valley is already lower than the K-valley at room temperature. Polarization increases with the number of WS_2_ layers because of the higher K–Λ energy difference. The Λ-valley thus determines the contrast between the high polarization in few-layer WS_2_ and low polarization for monolayer WS_2_. It also causes the contrast between few-layer WS_2_ and WSe_2_ at room temperature. The protection of polarization by the emergence of an indirect transition is a striking manifestation of interlayer interactions at the sub-nanometer scale. The control of the band structure and its indirect transitions by changing the interlayer distance (*e.g.*, using strain or pressure), tuning the band gap (*e.g.*, *via* electrical gating), or through hetero- or homostructures opens a route to manipulate the entanglement of the spin, valley, and layer indexes for actively tunable *valleytronics*.

## Author contributions

R.H.G., S.W., and A.G.C. designed the experiments. R.H.G. carried out the experiments assisted by S.W, while R.H.G. and T.V.R. analyzed the data. A.G.C. provided guidance during the experiments and analysis. All authors discussed the results. R.H.G. and A.G.C. wrote the manuscript with contributions from all authors.

## Conflicts of interest

There are no conflicts to declare.

## Supplementary Material

NR-014-D2NR04800K-s001
