# Supplementary material for: Impact of indirect transitions on valley polarization in WS2 and WSe2
Source: Nanoscale. 2022 Nov 21;14(47):17761–9. doi: 10.1039/d2nr04800k (PMC9730303; doi:10.1039/d2nr04800k)
Supplement: NR-014-D2NR04800K-s001 [file NR-014-D2NR04800K-s001.pdf]

Electronic Supplementary Material (ESI) for Nanoscale.
This journal is © The Royal Society of Chemistry 2022

Supplementary Information

Impact of indirect transitions on valley polarization in WS_2 and WSe_2

Rasmus H. Godiksen[1], Shaojun Wang[1,2], T.V. Raziman[1],

Jaime Gómez Rivas[1], Alberto G. Curto[1,3,4*]

[1]*Dep. Applied Physics and Institute for Photonic Integration,*

Eindhoven University of Technology, Eindhoven, The Netherlands

[2]*MOE Key Lab. of Modern Optical Technologies and Jiangsu Key Lab. of Advanced Optical Manufacturing Technologies, School of Optoelectronic Science and Engineering, Soochow University, Suzhou 215006, China*

[3]*Photonics Research Group, Ghent University-imec, Ghent, Belgium*

[4]*Center for Nano- and Biophotonics, Ghent University, Ghent, Belgium*

* Corresponding author: A.G.Curto@TUe.nl

Table of contents

Supplementary Section S1: Room temperature photoluminescence 2
  Resonant excitation of bilayer WSe_2 2
  Thickness-dependent polarization 2
Supplementary Section S2: Temperature-dependent photoluminescence 6
  Bilayer photoluminescence spectra 6
  Evidence of a dark ground state in bilayer WSe_2 8
  Temperature-dependent polarization in multilayer WSe_2 10

Supplementary Section S1: Room temperature photoluminescence

1. Resonant excitation of bilayer WSe_2

Throughout our study, we report polarization values measured with excitation at a constant photon energy of 2.040 eV, which is close to resonance for bilayer WSe_2 . Here, we check that bilayer WSe_2 does not show spin-valley polarization either under resonant excitation conditions (Supplementary Figure S1). We used an excitation energy of 1.681 eV, which is close to the WSe_2 bilayer K-K exciton emission around 1.62 eV.

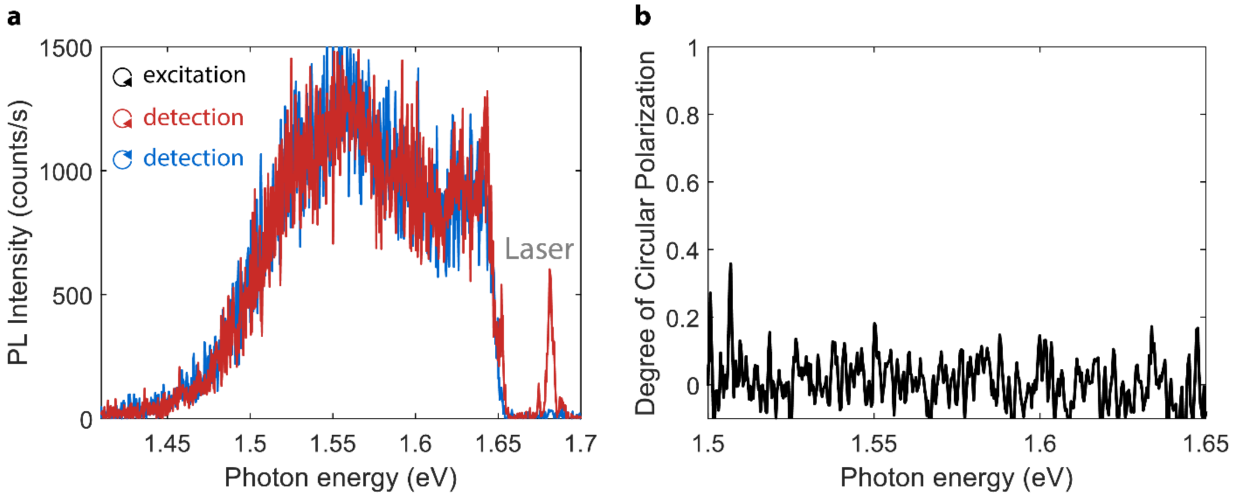

Supplementary Figure S1. a, Polarization-resolved PL spectrum for bilayer WSe_2 under near-resonant excitation (1.681 eV) at room temperature. **b,** Degree of circular polarization as a function of photon energy. No DOCP is measurable.

2. Thickness-dependent polarization

We determine the thickness of our WS_2 and WSe_2 samples by using a combination of reflection contrast microscopy, atomic force microscopy, and photoluminescence measurements. Similarly, for both WS_2 and WSe_2, the monolayers show bright emission due to their direct band gap. At room temperature, their emission spectrum shows a single peak. When increasing the thickness, a second peak emerges, which shifts to lower energy with an increasing layer thickness

(Supplementary Figure S2). Only WS_2 shows an increase in the DOCP with thickness, whereas in WSe_2 the emission remains unpolarized for all thicknesses when excited with 2.04 eV (Supplementary Figure S3) and 1.796 eV (Supplementary Figure S4).

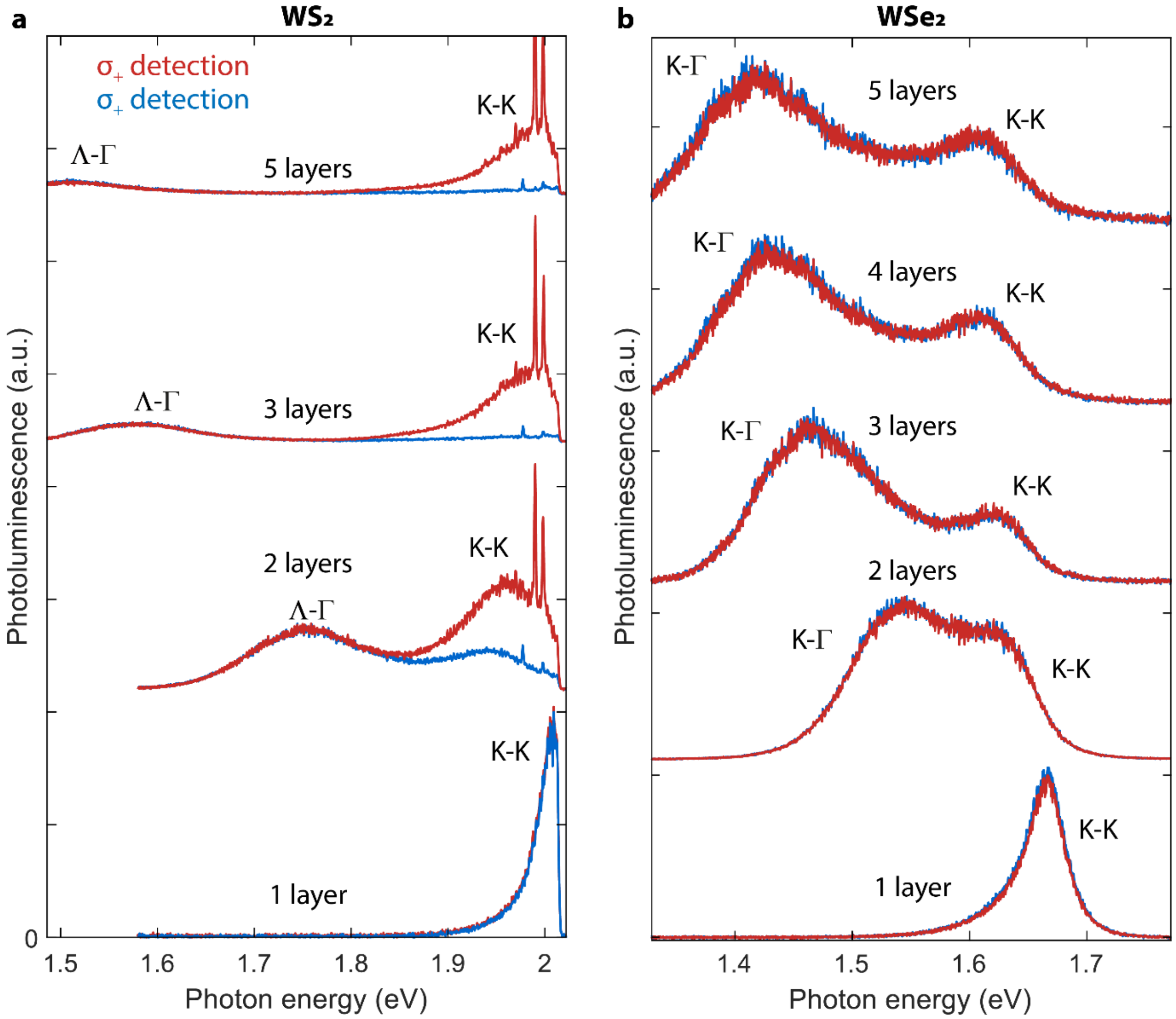

Supplementary Figure S2. Polarization-resolved PL spectra at room temperature for different thicknesses. **a,** WS_2. **b,** WSe_2. Spectra are vertically shifted by a constant for clarity.

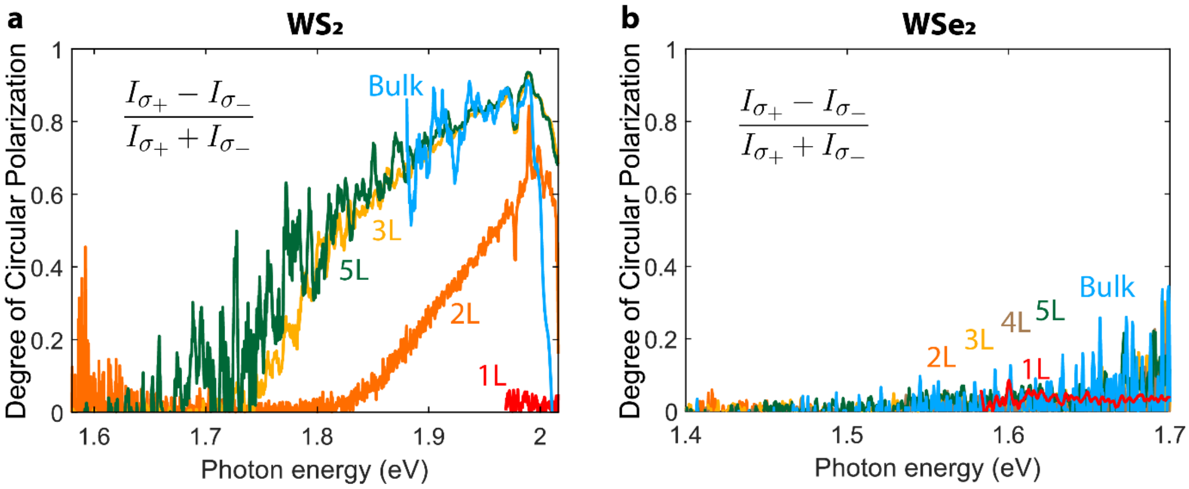

Supplementary Figure S3. Degree of circular polarization at room temperature as a function of emission wavelength for a set of different thicknesses for **a,** WS_2, and **b,** WSe_2.

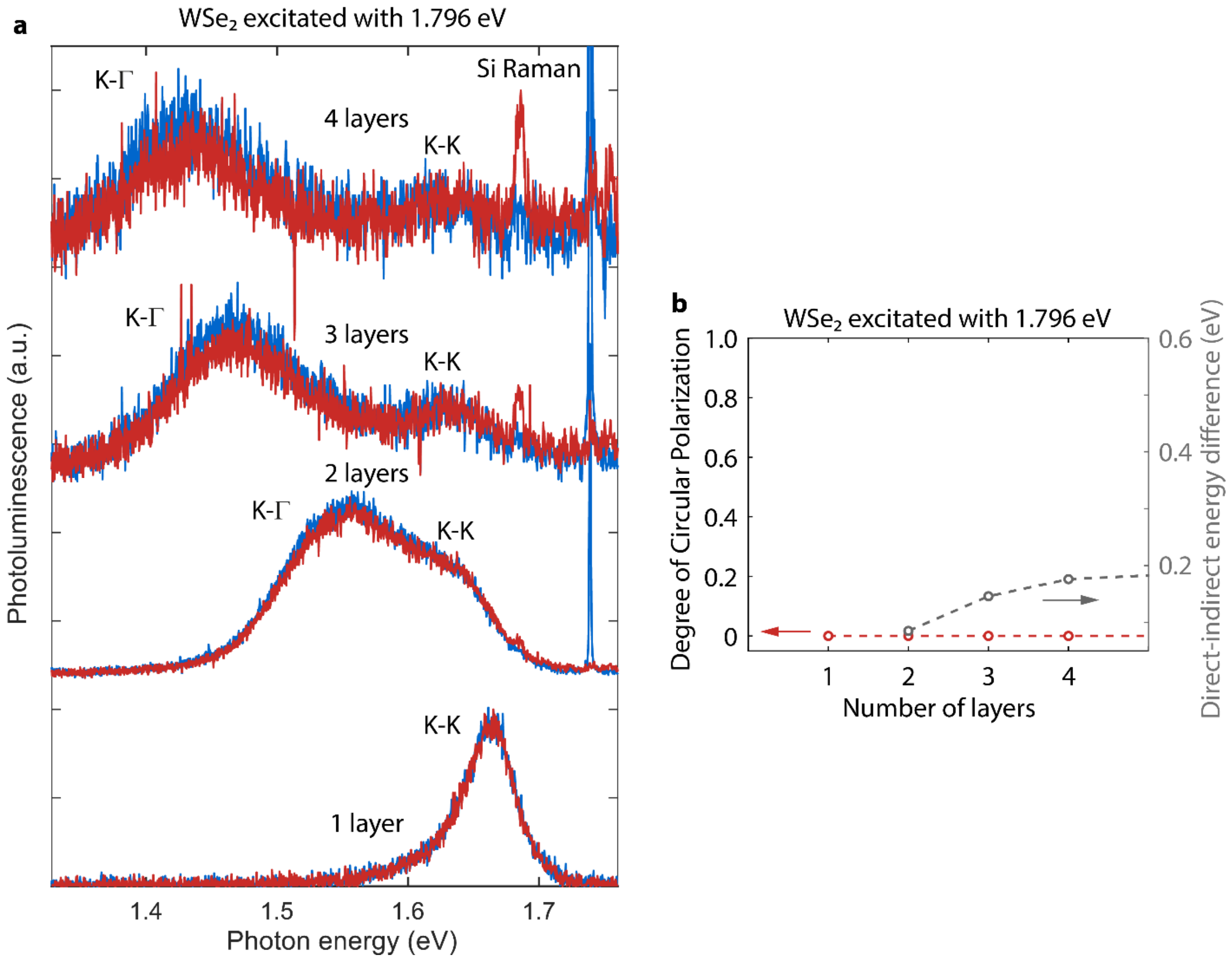

Supplementary Figure S4. Polarization-resolved PL spectra at room temperature for different thicknesses of WSe_2 excited with 1.796 eV. **a,** Thickness dependent spectra. **b,** Thickness vs the DOCP and the direct-indirect energy difference for the spectra in **a**.

Supplementary Section S2: Temperature-dependent photoluminescence

1. Bilayer photoluminescence spectra

When the temperature decreases, the two photoluminescence peaks shift with temperature (Supplementary Figure S4). In bilayer WS_2, the polarization also increases. However, in bilayer WSe_2, polarization only appears below $T = 160$ K (Supplementary Figure S5).

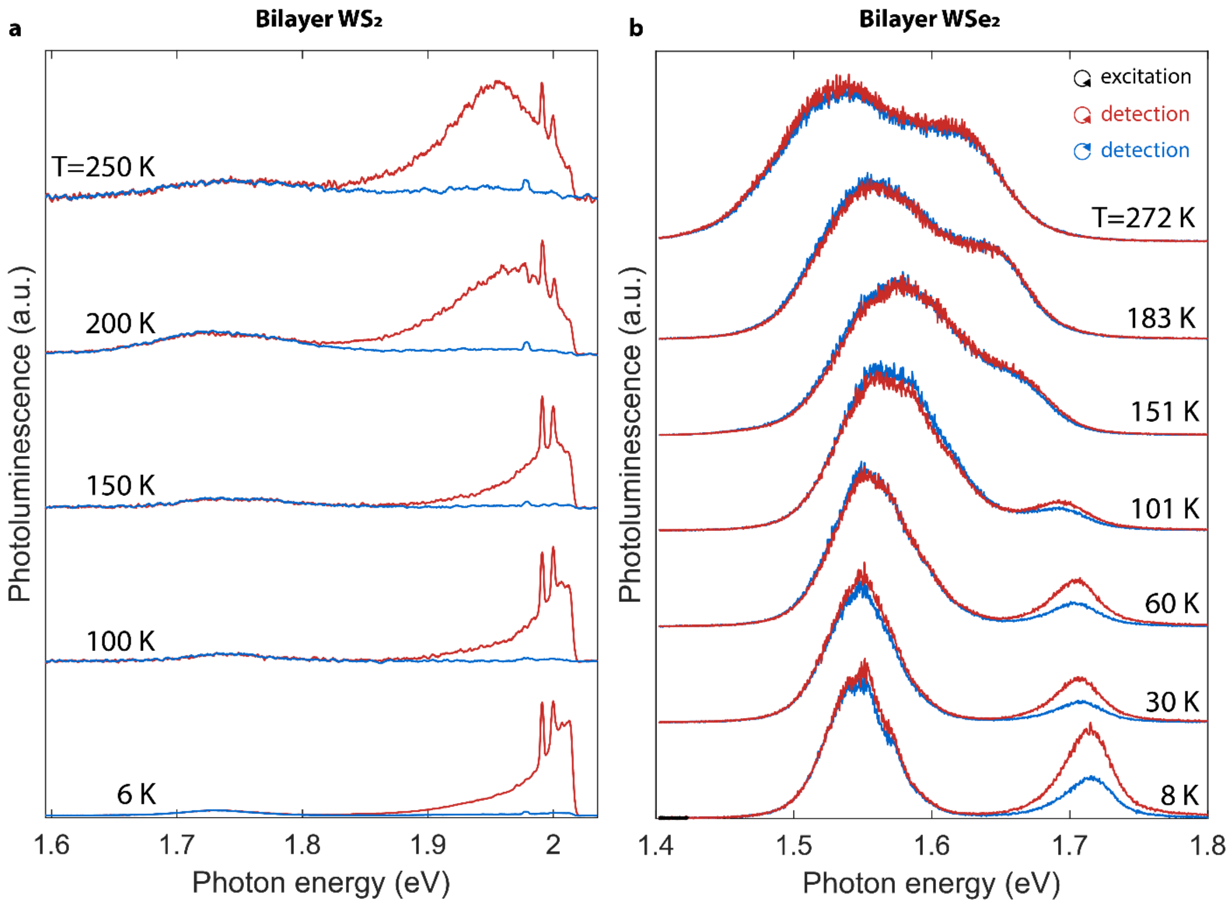

Supplementary Figure S5. Polarization-resolved PL spectra at different temperatures for bilayer samples. **a,** WS_2. **b,** WSe_2. Spectra are vertically shifted by a constant for clarity.

Supplementary Table S1. Fitting parameters obtained using Equation 2 in the main text in Figure 3. We contained the factor of 2 in the denominator of Equation 2 in the fitting parameter, *c*.

Material	*c*	ΔE (meV)
**WS_2**	0.12	74.6
**WSe_2**	0.076	72.5

2. Fitting using the O'Donnel equation

We fit the peak position as a function of temperature using two equations. Fitting using the Varshni equation1 was presented in the main text. Here, we fit the peak position using the O'Donnell equation[1]

$$E_g(T) = E_g(0) - S\langle\hbar\omega\rangle[\coth(\langle\hbar\omega\rangle/2k_BT) - 1] \qquad \text{(S1)}$$

where T is the temperature, $E_g(0)$ is the excitonic band gap, S is the Huang-Rhys factor, $\langle\hbar\omega\rangle$, is an average phonon energy, and k_B is the Boltzmann constant. The obtained fitting parameters are listed in Supplementary Table S2. Fitting using the O'Donnell equation yields as good a fit as with the Varshni equation, i.e., $R^2 = 0.9999$ when comparing the two fits. The main variation one might encounter between these two fitting methods will be expressed mainly in the range T=0-20 K, where we do not have several data points, as the band gap energy varies less in this range. The O'Donnell equation has a more profound theoretical background, and its fitting parameters are more well defined[1]. The Huang-Rhys factor, *S*, describes the exciton-phonon coupling strength of a certain transition. Comparing the values for each transition in Supplementary Table S2, we note that the exciton-phonon coupling strength is much larger for transitions that involve electrons in the K-valley compared to the Λ-valley. Similarly, the average phonon energy is also smaller for Λ-Γ excitons, suggesting that Λ-Γ excitons are more resistant to scattering by phonons.

Supplementary Table S2. Fitting parameters obtained using Equation S1 with the experimental data in Figure 3a-b.

Material / Transition		$E_g(0)$ (eV)	S (-)	$\langle \hbar\omega \rangle$ (meV)
**WS_2**	K-K	2.045	2.979	14.6
	Λ-Γ	1.737	0.997	2.0
**WSe_2**	K-K	1.713	2.957	16.5
	K-Γ	1.600	1.791	12.4
	Λ-Γ	1.546	0.991	6.3

3. Evidence of a dark ground state in bilayer WSe_2

In W-based monolayers, the dark excitons lie lower in energy than the bright excitons and transitions between the lowest conduction band and the top valence band at K is spin-forbidden (dark K-K exciton) due to spin splitting[2]. As evidence for bright-dark excitons in bilayer WSe_2, we observe a decrease of the K-K intensity with decreasing temperature consistent with reduced thermalization from dark to bright excitons[2–4] (Supplementary Figure S6). We fit the measured integrated PL intensity as a function of temperature to the expression $I_{PL}(T)/I_{PL}(0) - 1 = C\, exp(-E_D/k_B T)$, where $I_{PL}(T)$ is the measured intensity as a function of temperature, $I_{PL}(0)$ is the intensity at $T = 0$ K, C is a constant, k_B is the Boltzmann constant, and E_D is the characteristic energy barrier that defines the slope of the emission. From the fit, we obtain $E_D = 37.9$ meV, which is in good agreement with the bright-dark exciton splitting in monolayer WSe_2[5]. We expect a similar value for bilayer WSe_2 due to the limited effect of layer-layer interactions on the band structure near the K-point of the Brillouin zone.

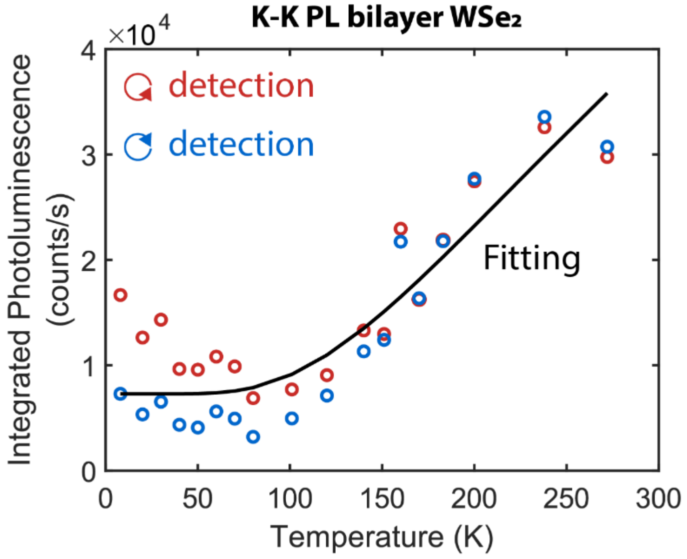

Supplementary Figure S6. Spectrally integrated PL of the A exciton emission as a function of temperature for both circular polarizations when excited with a 2.04 eV laser. The drop in emission intensity with temperature is consistent with a dark exciton ground state. The fitting is described in the text.

4. **Temperature-dependent polarization with varying thickness in WSe_2**

For a fixed temperature, if we increase the WSe_2 thickness to three or four layers, the K-Λ conduction band difference should become smaller. Similarly, the onset of an increase in DOCP should occur at a higher temperature compared to a bilayer. We confirm this trend by measuring the emission DOCP for three and four layers of WSe_2 and by comparing it as a function of temperature to that of a bilayer (Supplementary Figure S7).

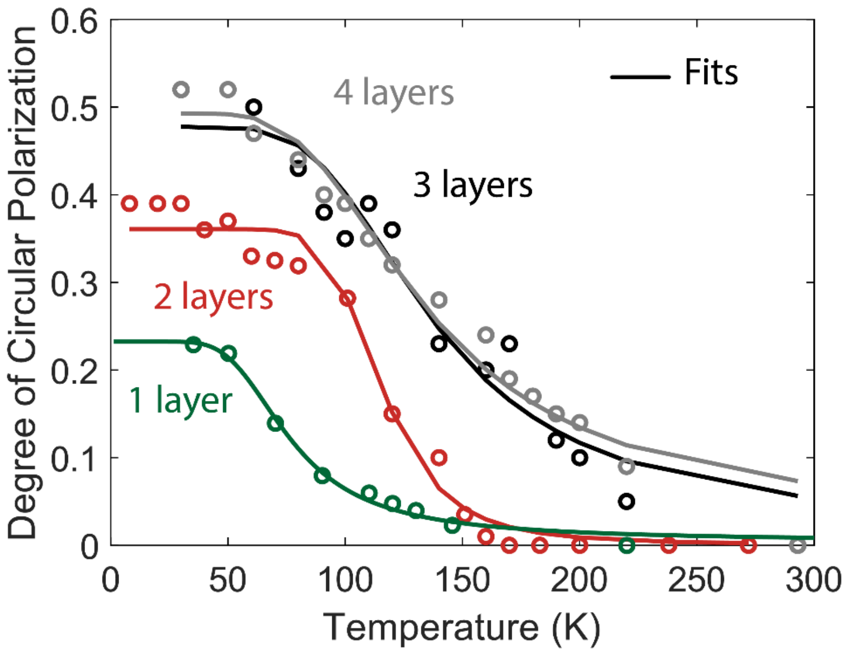

Supplementary Figure S7. Temperature-dependent DOCP measurements for 1, 2, 3, and 4 layers of WSe_2 showing an increase in the onset temperature of DOCP with increasing layer thickness. The 2, 3, and 4 layer data was acquired using 2.04 eV excitation. The monolayer data was acquired using 1.796 eV excitation. The fits are made by assuming a Boltzmann distribution for the K-K' intervalley scattering, see details in the main text.

References

1. O'Donnell, K. P. & Chen, X. Temperature dependence of semiconductor band gaps. *Appl. Phys. Lett.* **58**, 2924 (1991).

2. Zhang, X.-X., You, Y., Yang, S., Zhao, F. & Heinz, T. F. Experimental Evidence for Dark Excitons in Monolayer WSe_2. *Phys. Rev. Lett.* **115**, 257403 (2015).

3. Malic, E. *et al.* Dark excitons in transition metal dichalcogenides. *Phys. Rev. Mater.* **2**, 014002 (2018).

4. Zhang, M., Fu, J., Dias, A. C. & Qu, F. Optically dark excitonic states mediated exciton and biexciton valley dynamics in monolayer WSe_2. *J. Phys. Condens. Matter* **30**, 265502 (2018).

5. Wang, G. *et al.* In-Plane Propagation of Light in Transition Metal Dichalcogenide Monolayers: Optical Selection Rules. *Phys. Rev. Lett.* **119**, 047401 (2017).
